# Datasets from label-free quantitative proteomic analysis of human glomeruli with sclerotic lesions

**DOI:** 10.1016/j.dib.2015.05.013

**Published:** 2015-05-27

**Authors:** Ying Zhang, Bo Xu, Naohiko Kinoshita, Yutaka Yoshida, Masayuki Tasaki, Hidehiko Fujinaka, Sameh Magdeldin, Eishin Yaoita, Tadashi Yamamoto

**Affiliations:** a Department of Structural Pathology, Institute of Nephrology, Graduate School of Medical and Dental Sciences, Niigata University, Niigata, Japan; b Biofluid Biomarker Center (BB-C), Institute for Research Collaboration and Promotion, Niigata University, Niigata, Japan; c Division of Urology, Department of Regenerative and Transplant Medicine, Graduate School of Medical and Dental Sciences, Niigata University, Niigata, Japan; d Institute of Clinical Research, Niigata National Hospital, Kashiwazaki, Japan; e Department of Physiology, Faculty of Veterinary Medicine, Suez Canal University, Ismailia, Egypt

## Abstract

Human glomeruli with intermediate (i-GS) and advanced (GS) sclerotic lesions as well as the normal control (Nor) were captured from laser microdissection, digested by trypsin and subjected to shotgun LC-MS/MS analysis (LTQ-Orbitrap XL). The label-free quantification was performed using the Normalized Spectral Index (*SI*_*N*_) to assess the relative molar concentration of each protein identified in a sample. All the experimental data are shown in this article. The data is associated to the research article submitted to Journal of Proteomics [1].

**Specifications Table**

**Table:** 

Subject area	Chemistry, Biology
More specific subject area	Label-free quantitative proteomics
Type of data	a. *Excel datasheets with peptide and protein identifications in each sample.* b. *Excel datasheet with Normalized Spectral Index* (*SI*_*N*_) *of the proteins identified in each sample.* c. *Venn diagrams for peptide*/*protein identifications in each group.* d. *Graphs for comparison of SI*_*N*_ *values between the sample groups.*
How data was acquired	MS/MS data acquired from LTQ-Orbitrap XL (Thermo Scientific) combined with nanoscale C18 reversed phase liquid chromatography (DiNa-A, KYA technologies).
	Peptide/protein identification results exported from Mascot.
Data format	.xlsx (peptide/protein identifications and *SI*_*N*_ values)
	.PDF (Venn diagrams and comparison of *SI*_*N*_ values)
Experimental factors	No sample pretreatment applied
Experimental features	Human glomerular sections were collected by laser microdissection and digested by trypsin. The peptide sample was purified with StageTips C18 before analyzed by LC-MS/MS in triplicates.
Data source location	Niigata City, Japan
Data accessibility	All the experimental data are available in this article.

**Value of the data**

• In-depth proteomic profiles provide comprehensive protein composition of human sclerotic glomeruli.
• *SI*_*N*_-based label-free quantitative datasets are useful to explore the key biological events which would be critically involved in the progression of human glomerulosclerosis.
• Detailed information on peptide-spectrum matches and ion intensities for spectral queries enables further bioinformatic analysis.

## 1 Data, experimental design, materials and methods

We aimed to characterize human glomeruli with intermediate and advanced sclerotic lesions by label-free quantitative proteomic approach in combination with laser microdissection. Macroscopically normal kidney tissues were obtained from patients who underwent nephrectomy due to urological cancers. Sclerotic glomeruli, which were excluded from specific renal diseases and assumed to be aging-related, were divided into two groups, intermediate (i-GS) and advanced (GS) sclerosis, as well as the normal control (Nor). Glomerular sections (10 μm–thick, fixed by methyl–Carnoy) were collected by laser microdissection (50 sections/sample, 3 samples/group from 3 patients). The detailed information on patients and specimens are given in the associated article submitted to Journal of Proteomics [1].

Each glomerular sample was directly digested with 15 μl of activated trypsin solution (20 ng/μl) at 37 °C overnight. After trypsin digestion, 1 μl of 50% trifluoacetic acid (TFA) was added to the peptide mixture to quench the trypsin activity. Peptides were eluted and purified using StageTips C18 (Thermo Scientific) according to the manual instructions [2]. Briefly, the C18 tips were firstly activated with solvent A (80% acetonitrile, 5% formic acid) and re-equilibrated with solvent B (5% formic acid) before sample loading. Then the peptides were eluted twice with 20 μl of solvent A. Finally, the peptide eluate was dehydrated in a Speedvac for dryness and stored at −30 °C until LC-MS/MS analysis. We estimated that around 1 μg of peptides could be extracted from one sample according to BCA assay. As the peptide yield is very limited, we did not perform BCA assay for the samples which were analyzed by LC-MS/MS. All peptides extracted from each sample were directly analyzed in triplicate.

**Fig. 1:**
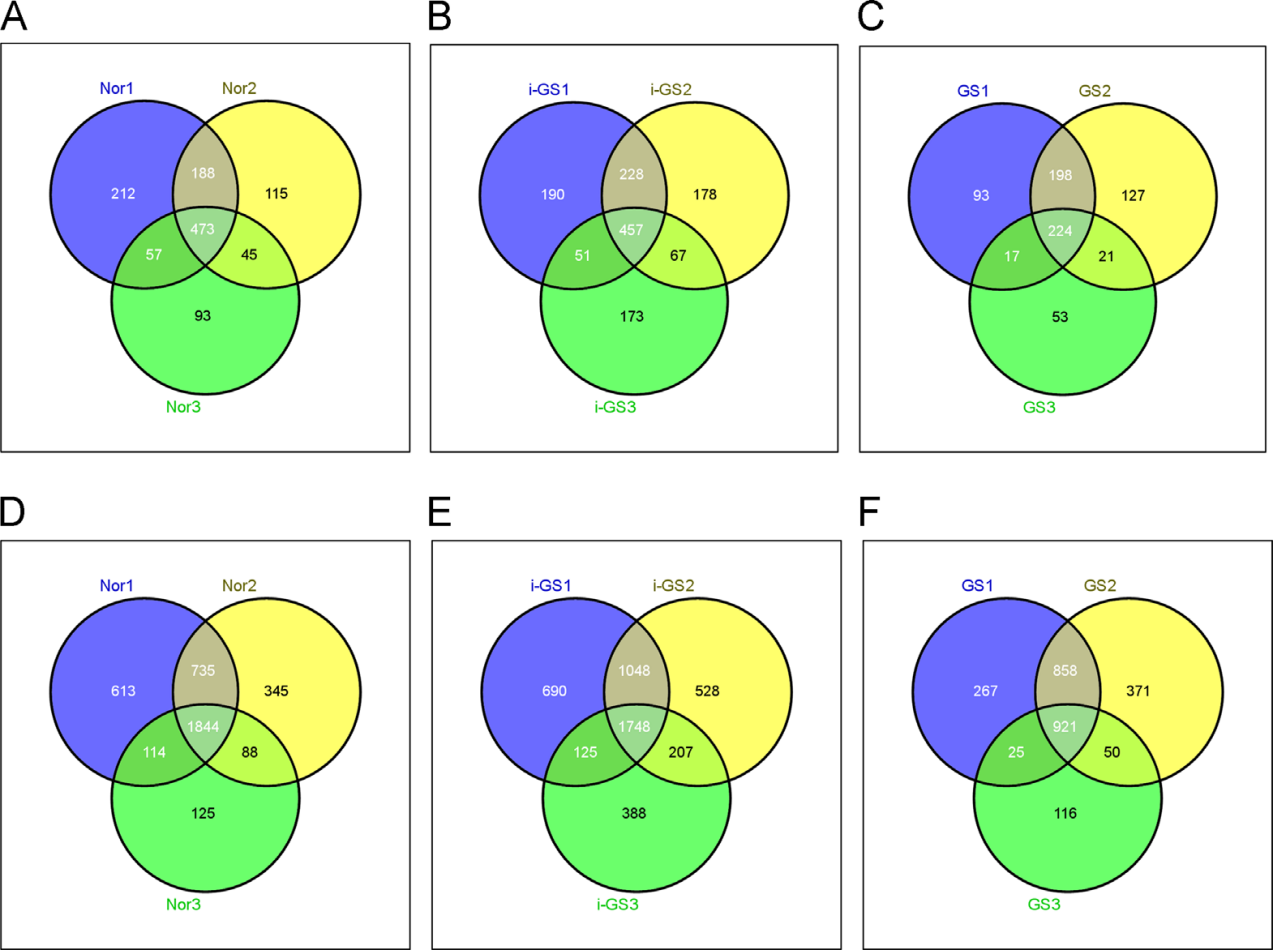
Reproducibility of protein and peptide identifications by LC-MS/MS analysis in three biological duplicates of each group. A, B and C. Venn diagrams for the proteins identified in three biological samples of the groups Nor, i-GS and GS, respectively. D, E and F. Venn diagrams for the peptides identified in three biological samples of the groups Nor, i-GS and GS, respectively.

Each peptide sample was solubilized in the sample solution (2% acetonitrile, 0.1% formic acid) and measured in triplicates by LTQ-Orbitrap XL (Thermo Scientific) combined with nanoscale C18 reversed phase liquid chromatography (DiNa-A, KYA technologies). The peptides were separated on a C18 separation column (75 μm×100 mm, particle size of 3 μm, pore size of 120 Å) and eluted with a 120 min mobile phase gradient at the flow rate of 300 nl/min. MS survey scan (m/z 350–1600, resolution 60,000) was acquired in the Orbitrap and the five most intensive precursor ions were fragmented in linear ion trap. The dynamic exclusion time was set to 60 s. The MS/MS spectral data obtained from the triplicate measurements for each sample were merged by Mascot Daemon software (Matrix Science) and then searched against UniProtKB/Swiss-Prot human database (release 2014_04) using the Mascot search engine (Version 2.3.01). The parameters for protein identification used were as follows: peptide tolerance, 10 ppm; MS/MS tolerance, 0.8 Da; fixed modification, none; variable modification, oxidation on methionine (M), histidine (H), and tryptophan (W); No. of missed trypsin cleavages, 2; significance threshold *p*<0.01; protein scoring, MudPIT (multidimensional protein identification technology). Peptide FDR was controlled <1%. Protein hits with two matched peptides were considered as confident identifications. To eliminate protein redundancy, proteins with different accession numbers but same gene name were grouped and the protein with the highest score was selected to generate the final protein identification list. The reproducibility of peptide and protein identifications among three samples in each group are examined by Venn diagrams (Fig. 1).

The label-free quantitative proteomic analysis was performed using Normalized Spectral Index (*SI*_*N*_) based on the previous description [3,4]. This label-free quantitative value, *SI*_*N*_, consists of multiple MS abundance features for a given protein hit: peptide count, spectral count for all assigned peptides, total ion intensities of all matched MS/MS spectra, and protein length (number of amino acids). Firstly, the abundance of a protein is calculated as the cumulative ion intensities of all assigned MS/MS spectra for a protein hit. Secondly, the intensity of the protein is normalized by dividing its intensity by the total intensities of all proteins identified in the dataset. Thirdly, the normalized intensity of this protein is divided by the protein length to obtain the relative molar concentration of this protein in the sample. In this study, we grouped the identified proteins according to their gene names, then filtered out the duplicate spectra occurring in the same group, and finally calculated the *SI*_*N*_ value for each protein group using the following formula:

$$(SI_{N})j=\frac{SI_{j}/\sum_{i=1}^{n}SI_{i}}{L_{j}}\;$$

(*SI*_*N*_)_*j*_: Normalized spectral index of the protein group with gene name *j*.
*SI*_*j*_: Total ion intensities of MS/MS spectra assigned to the protein group with gene name j.
$\sum_{i=1}^{n}\;$*SI*_*i*_:Total ion intensities of all the protein groups identified in a given sample.
*L*_*j*_:The length of the protein having the highest protein score in the group with gene name *j*.

*SI*_*N*_ calculation was performed using a home-made Excel VBA script. Mascot search results of detailed peptide/protein identification in each sample, e.g. peptide-spectrum matches, protein hits, and ion intensities of MS/MS spectral queries, etc. are shown in Supplementary Table 1. In this table, the proteomic data necessary for *SI*_*N*_ calculation are highlighted in blue. The *SI*_*N*_ values of identified proteins among three samples in each group and the results of statistical analysis are shown in Supplementary Table 2.

**Fig. 2:**
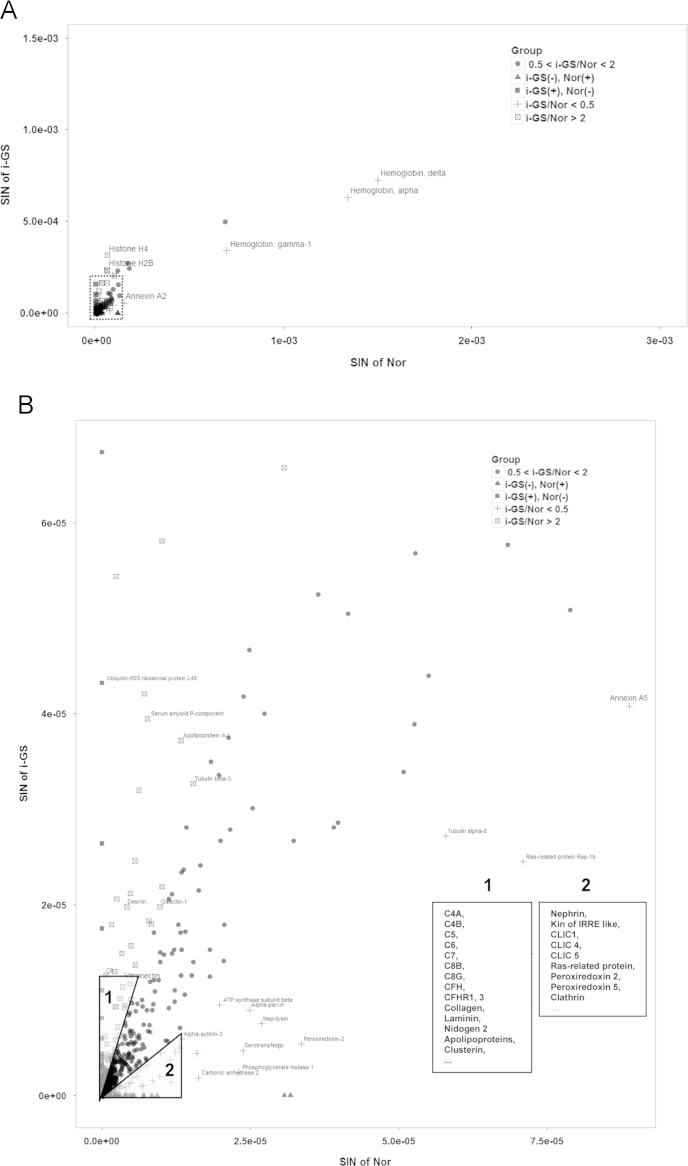
Label-free quantification (Normalized Spectra Index, SI_N_) and comparison of the proteins identified in i-GS and Nor. A. The protein distribution pattern in the whole SI_N_ dynamic range. The narrower SI_N_ range outlined with a dotted line is magnified in B. B. Enlarged image for the proteins in the narrower SI_N_ range in A. The representative proteins increased and decreased in GS in the fields 1 and 2 are listed below the symbol legend.

**Fig. 3:**
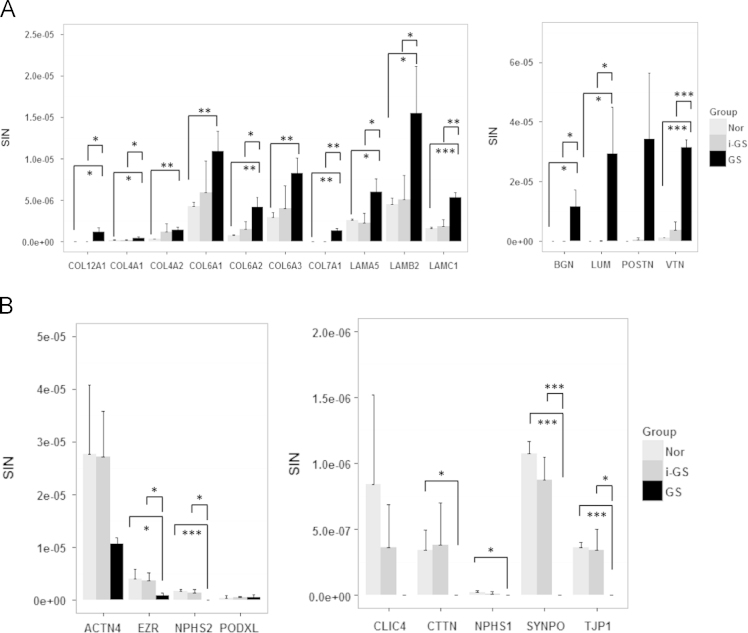
Proteins involved in extracellular matrix construction are significantly increased in glomerular sclerosis while podocyte featured proteins are decreased. A. Extracellular matrix proteins. B. Podocyte slit diaphragm and cytoskeleton proteins. COL12A1, collagen, type XII, alpha 1; COL4A1, collagen, type IV, alpha 1; COL4A2, collagen, type IV, alpha 2; COL6A1, collagen, type VI, alpha 1; COL6A2, collagen, type VI, alpha 2; COL6A3, collagen, type VI, alpha 3; COL7A1, collagen, type VII, alpha 1; LAMA5, laminin, alpha 5; LAMB2, laminin, beta 2 (laminin S); LAMC1, laminin, gamma 1; BGN, biglycan; LUM, lumican; VTN, vitronectin; POSTN, periostin; ACTN4, actinin, alpha 4; EZR, ezrin; PODXL, podocalyxin; NPHS2, podocin; SYNPO, synaptopodin; CLIC4, chloride intracellular channel protein 4; TJP1, tight junction protein 1; CTTN, cortactin; NPHS1, Nephrin. Results are indicated as Mean±SD. *p<0.05; **p<0.01; ***p<0.001.

The *SI*_*N*_ values of each protein in the three samples of the groups i-GS and Nor were summed, compared and plotted in Fig. 2. The data suggest that complement components (C4, C5, C6, C7, C8, C9) and their regulators (CFH, CFHR1, vitronectin, clusterin) are at least twice increased in i-GS than Nor. More importantly, the over-expression of complement pathway indicates a general up-going tendency from i-GS to GS (see Ref. [1], Figs. 3 and 4). Additionally, diverse extracellular matrix proteins are found predominantly accumulated in sclerotic glomeruli whereas various podocyte proteins important to slit diaphragm and cytoskeletal integrity are dramatically reduced or lost (Fig. 3).

## Funding sources

This work was supported by Grant-in-Aid for Young Scientists B (15K19448) to Y.Z. from Japan Society for the Promotion of Science, Grant-in-Aid for Publication of Scientific Research Results (228071) to T.Y. from Ministry of Education, Culture, Sports, Science and Technology in Japan and Grant-in-aid for Diabetic Nephropathy and Nephrosclerosis Research to T.Y. from the Ministry of Health, Labor and Welfare of Japan.
